# A mitochondria-targeted coenzyme Q peptoid induces superoxide dismutase and alleviates salinity stress in plant cells

**DOI:** 10.1038/s41598-020-68491-4

**Published:** 2020-07-14

**Authors:** Kinfemichael Geressu Asfaw, Qiong Liu, Xiaolu Xu, Christina Manz, Sabine Purper, Rose Eghbalian, Stephan W. Münch, Ilona Wehl, Stefan Bräse, Elisabeth Eiche, Bettina Hause, Ivan Bogeski, Ute Schepers, Michael Riemann, Peter Nick

**Affiliations:** 1 Molecular Cell Biology, Botanical Institute, Karlsruhe Institute of Technology (KIT), Fritz-Haber-Weg 4, D-76131 Karlsruhe, Germany; 2 Institute of Organic Chemistry, Organic Chemistry I, Karlsruhe Institute of Technology (KIT), Fritz-Haber-Weg 6, D-76131 Karlsruhe, Germany; 3 Molecular Physiology, Institute of Cardiovascular Physiology, University Medical Center, Georg-August-University, 37073 Göttingen, Germany; 4 Institute of Biological and Chemical Systems-Functional Molecular Systems (IBCS-FMS), Karlsruhe Institute of Technology (KIT), Hermann-von-Helmholtz-Platz 1, D-76344 Eggenstein-Leopoldshafen, Germany; 5 Institute of Functional Interfaces (IFG), Karlsruhe Institute of Technology (KIT), Hermann-von-Helmholtz-Platz 1, D-76344 Eggenstein-Leopoldshafen, Germany; 6 Institute of Applied Geochemistry (AGW), Geochemistry and Economic Geology Group, Karlsruhe Institute of Technology (KIT), Adenauerring 20b, D-76131 Karlsruhe, Germany; 7 Department of Cell and Metabolic Biology, Leibniz Institute of Plant Biochemistry, Weinberg 3, D-06120 Halle (Saale), Germany

**Keywords:** Fluorescence imaging, Energy metabolism, Salt

## Abstract

Salinity is a serious challenge to global agriculture and threatens human food security. Plant cells can respond to salt stress either by activation of adaptive responses, or by programmed cell death. The mechanisms deciding the respective response are far from understood, but seem to depend on the degree, to which mitochondria can maintain oxidative homeostasis. Using plant PeptoQ, a Trojan Peptoid, as vehicle, it is possible to transport a coenzyme Q10 (CoQ10) derivative into plant mitochondria. We show that salinity stress in tobacco BY-2 cells (*Nicotiana tabacum* L. cv Bright Yellow-2) can be mitigated by pretreatment with plant PeptoQ with respect to numerous aspects including proliferation, expansion, redox homeostasis, and programmed cell death. We tested the salinity response for transcripts from nine salt-stress related-genes representing different adaptive responses. While most did not show any significant response, the salt response of the transcription factor NtNAC, probably involved in mitochondrial retrograde signaling, was significantly modulated by the plant PeptoQ. Most strikingly, transcripts for the mitochondrial, Mn-dependent Superoxide Dismutase were rapidly and drastically upregulated in presence of the peptoid, and this response was disappearing in presence of salt. The same pattern, albeit at lower amplitude, was seen for the sodium exporter SOS1. The findings are discussed by a model, where plant PeptoQ modulates retrograde signalling to the nucleus leading to a strong expression of mitochondrial SOD, what renders mitochondria more resilient to perturbations of oxidative balance, such that cells escape salt induced cell death and remain viable.

## Introduction

By the year 2050, world agriculture should be able to boost production of food crops by 70% to feed the projected 9.1 billion people^1^. Salt stress is one of the major challenges to this effort and has already affected 20% of cultivated land worldwide. Global warming and suboptimal irrigation accentuate the problem^2,3^. The molecular, cellular, and physiological mechanisms underlying the severe effect of salinity on plant growth, development and yield have been reviewed comprehensively^3–5^. Based on their response to salt stress, plants are categorised into the tolerant halophytes and the sensitive glycophytes. Unfortunately, most of the crop species belong to the glycophytes^2,3^. To address the plant response to salinity, it has been useful to reduce complexity by addressing the osmotic, ionic, and oxidative component of salt stress separately^4^.

Osmotic stress (hyperosmotic stress) is considered as central component of salinity. It causes cellular damage, such as membrane disruption, imbalanced oxidative homeostasis, for instance, by non-functional electron transport across the inner mitochondrial membrane (in green tissue also across the thylakoid), by impaired scavenging of reactive oxygen species (ROS), or by inhibition of antioxidant enzymes. This is accompanied by perturbed physiology, such as low root water conductivity, reduced growth rate^5^, or decreased photosynthetic activity because stomata close in consequence of low turgor^3^. Although the osmotic component is shared between salt and drought stresses^4^, the response of the plant, while sharing some events, differs even qualitatively as shown by comparative analysis in rice, where the osmotic component of water scarcity and salinity stress was kept constant^6^.

The second component of salt stress is ionic stress (hyperionic stress), caused by the accumulation of Na^+^ and Cl^−^ ions that can enter the plant cell, in case of sodium, through non-selective cation channels, and activate a whole plethora of damage-related, but also adaptive, cellular response^3–5^. The challenged cell will either adapt, for instance, by sequestering the sodium ions into the vacuole, or it will activate salt-dependent necrosis. This response, while being lethal for the individual cell, may be adaptive on the level of the whole plant, because necrotic organs can be shed, such that salt is removed and the young tissues are protected, especially the meristems, from which new organs can be generated, once the stress episode is overcome^7^. The decision between these two response patterns depends on the relationship in the temporal pattern of the individual responses (reviewed in^8^). The third component of salinity stress is oxidative stress, resulting from uncontrolled accumulation of reactive oxygen species (ROS), such as superoxide radical (O_2_^**∙**−^), or hydrogen peroxide (H_2_O_2_). The generation of these ROS in different sites of the cell, the molecular mechanisms of their genesis and control, and the responses to balanced and perturbed ROS levels, have been reviewed repeatedly^4,9–12^. During the stress response of plant cells, ROS can play a double role—on the one hand, they can accumulate in consequence of stress-induced perturbation of cellular homeostasis; on the other hand, they are central for the signalling that activates cellular adaptation to stress. This functional duality of ROS, although reviewed comprehensively^13^ raises the issue of specificity. Depending on the subcellular location, ROS trigger quite different events. For instance, the plasma-membrane located NADPH oxidase, Respiratory burst oxidase Homolog (RboH) has emerged as central source for signalling ROS^14^. The apoplastic ROS generated by RboH trigger a signal that is conveyed through calcium influx^15^, G-protein dependent phospholipase D signalling^16^, and mitogen-activated protein kinase (MAPK) cascades^17^ culminating in the activation of transcription factors of the WRKY family that will, among other targets, also amplify the expression of RboH itself ^18^.

In contrast, the ROS generated in consequence of perturbed mitochondrial function, will leak out through the permeability transition pore^19^, and convey a signal involving activity of the cyclin-dependent kinase CDKE^20^, converging on proteolytic cleavage of specific members of the NAC transcription factor family, such as ANAC013^21^, located in the ER, such that the released fragment will be imported into the nucleus and activate expression of genes harbouring a mitochondrial dysfunction motif in their promoters^22^. This so called retrograde signalling (because it moves from the mitochondria towards the nucleus) is expected to be central for non-photosynthetic tissue. Here, a perturbed mitochondrial electron transport chain is the central source of oxidative damage, especially in roots, where ROS accumulation in consequence of disbalanced photosynthesis does not play a role.

When fairly general small molecules, such as ROS, can evoke quite different cellular responses, depending on their subcellular localisation (for instance, apoplast versus mitochondria), it might be this subcellular localisation that confers specificity. To experimentally address the hypothesis that the information conveyed by a given molecule depends on the spatial coordinates of this molecule would require that the accumulation of ROS can be controlled differentially, depending on the respective intracellular region. Although it is possible to modulate steady-state levels of ROS by scavenging enzymes such as superoxide dismutase (SOD), or catalase (CAT), as well as by non-enzymatic antioxidants such as ascorbate or tocopherols^23^, these modulations are global, acting on the entire cell in the first place. To scavenge ROS in specific sites of the cell (for instance, in the mitochondria), leaving their accumulation in other sites of the cell (for instance, in the apoplast) untouched, is a task that is more challenging. Genetic engineering or adding inhibitors, are by their very nature scalar approaches and cannot be used in a straightforward manner to address spatial patterns of signals (although it would be possible, in principle, to achieve this goal by expression of scavenging enzymes under control of appropriate signal peptides). To modulate steady-state levels of ROS by targeting antioxidants to a specific target site within the cell would be more straightforward. However, such a strategy based on chemical engineering would require molecular vehicles that can pass membrane barriers and carry their functional cargo to the site of interest.

To this effect, cell penetrating peptides (CPPs) or protein transduction domains (PTDs) that can carry functional cargoes and pass across the plasma membrane have attracted considerable attention^24,25^. Using this approach in mammalian systems, proteins, plasmids, peptides, nucleic acids, siRNA, liposomes, and nanoparticles have been delivered effectively^26,27^. In plants, even though reports on their application are quite sporadic^28^, their uptake by protoplasts derived from tobacco suspension cell and Triticale mesophyll protoplasts has been achieved^29,30^. Although CPPs are effective in vitro, their bioavailability in vivo is limited due to hydrolysis by proteases. Thus, peptido-mimetics such as peptoids could serve as excellent alternative due to their high stability in vivo ^31^. Unlike CPPs, the side chain in peptoids (oligo-*N*-alkylglycines) is linked to the amide nitrogen instead of α carbon^32,33^. As a result, these peptoids lack hydrogen-bonding potential, which in turn prevents aggregation that would originate otherwise from their backbone, such that bioavailability is increased^32^. Contrary to CPPs, peptoids are insensitive to proteases and other modifying enzymes^34,35^. Furthermore, it is possible to produce chemically distinct peptoid libraries easily, cheaply and without the need for protecting groups unlike the case in CPPs, owing to the presence of structurally diverse amines^33^. Peptoids have already been shown to enter walled plant cells^31^. As next step, this tool could be used to deliver functional cargoes to specific intracellular targets.

**Figure 1:**
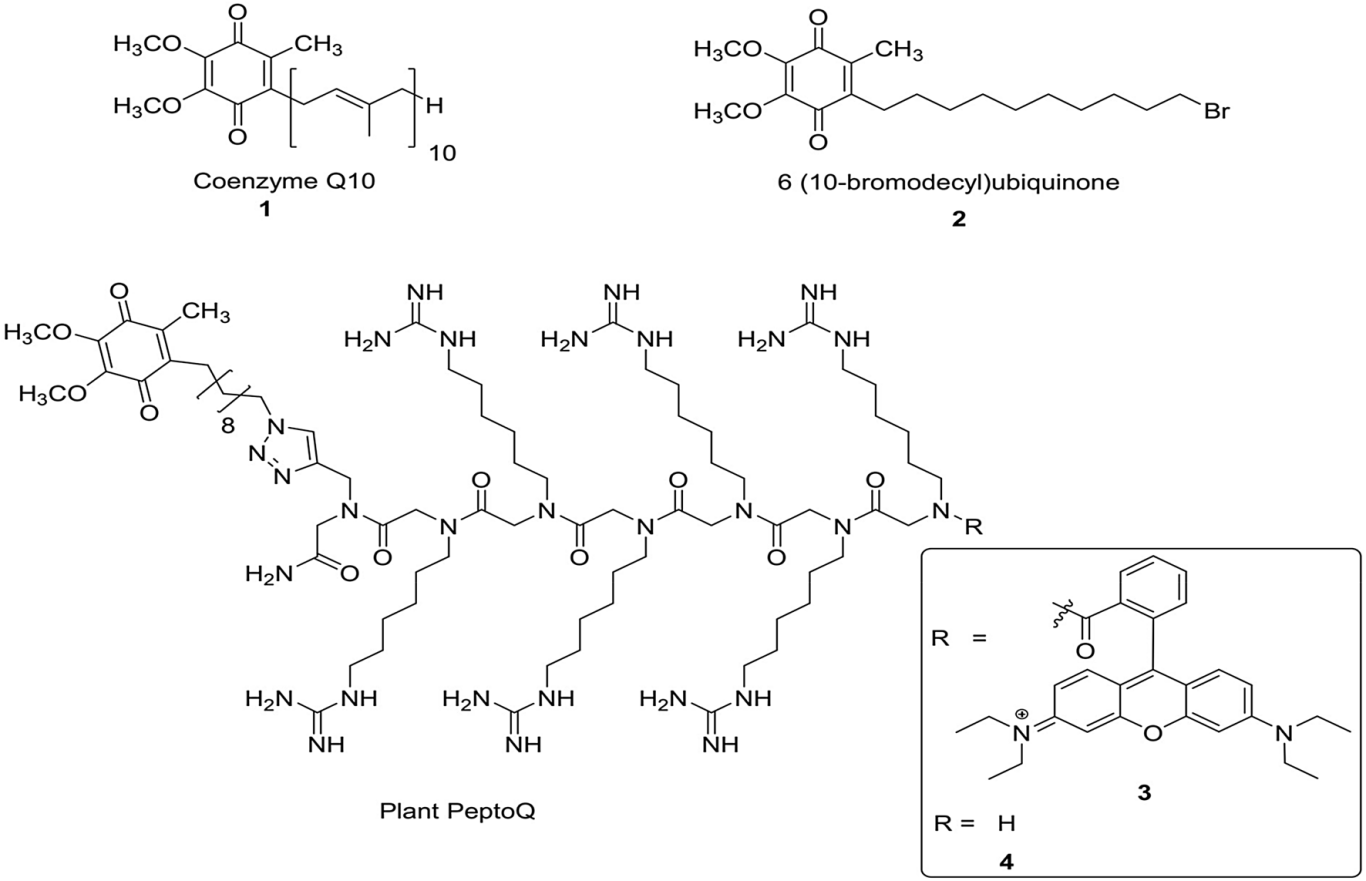
The chemical structures of CoQ10 **1,** the CoQ10 analogue 6-(10-bromodecyl) ubiquinone** 2** and the rhodamine-labelled and unlabelled plant PeptoQ** 3** and **4**.

In our previous work, we have shown that plant PeptoQ, a cell penetrating peptoid with a mitochondria-targeting motif and endowed with a semiquinone mimetic of coenzyme Q10 (Fig. 1), was rapidly and efficiently targeted to the mitochondria of walled tobacco BY-2 cells^36^ and conferred improved resilience to salt stress. Hence, in our current study, we analyse the mode of action behind this mitigation. We show that in the absence of the peptoid, cellular physiology (such as cell proliferation, cell expansion, viability, ion balance, and oxidative balance) proceed normally. However, when challenged by salt stress, the physiological homeostasis turns out to be more robust after treatment with plant PeptoQ. We asked the question, whether this improved resilience is linked with altered expression levels of candidate genes reporting the status of known adaptive responses. We found that the effect of plant PeptoQ correlates with elevated expression of mitochondrial superoxide dismutase.

## Results

### Plant PeptoQ mitigates the impact of salt stress on cell proliferation, cell expansion, and viability

Tobacco BY-2 cells display a sequence of cellular events over the progression of each culture cycle^37^: following a lag phase of around one day, needed for the migration of the nucleus from the lateral wall into the cell center, a proliferation phase follows, where the cells undergo 3–4 cycles of division. Around day 3 after subcultivation, proliferation comes to a halt, and the cells expand by uptake of water into their central vacuole. This expansion continues till the end of the culture cycle (lasting 7 days). Since the physiological state of the cells is clearly different during proliferation and expansion, which is already evident from the impressive expansion of the central vacuole seen during the expansion phase, we analysed the effect of peptoid treatment for both cellular events separately. In all experiments described in this study, the cells were pretreated with either 2 µM of plant PeptoQ or with culture medium without the peptoid as negative control for 2 h. We imposed two different levels of salt stress: 150 mM NaCl which generates a significant osmotic stress with a water potential of − 7.35 bars, while 75 mM NaCl represents a moderate stress level with a water potential of − 3.68 bars, which still would be more negative than the − 1.7 to − 2.5 bars found in expanding cells^38^.

**Figure 2:**
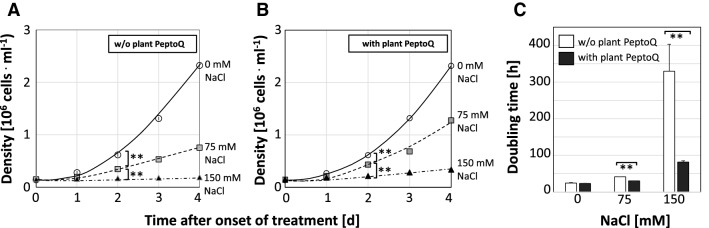
Effect of the plant PeptoQ on salt-induced inhibition of cell proliferation in tobacco BY-2 cells. Time courses of cell density in the absence of salt stress (0 mM NaCl, open circles), under moderate (75 mM NaCl, grey squares), and under severe salt stress (150 mM NaCl, black triangles) without (**A**) or with (**B**) plant PeptoQ (2 µM). (**C**) Doubling time estimated from the time constant of exponential growth over the concentration of NaCl without (open bars) or with (filled bars) plant PeptoQ (2 µM). Data represent mean values and standard errors of three independent experimental series. **Indicate differences significant at *P* ≤ 0.01 based on a Student’s *t* test.

Salinity caused a drastic decline in proliferation activity, and this decline was mitigated by pretreatment with 2 µM of plant PeptoQ (Fig. 2): Even for moderate salt stress (75 mM NaCl), cell density was reduced as compared to the control (Fig. 2A). This decrease was already significant (29%) at the first measured time point (24 h), and became progressively accentuated with time, with 44% inhibition at 48 h, 60% at 72 h, and 68% at 96 h. After pretreatment with 2 µM of plant PeptoQ, this decrease of cell density was significantly dampened (Fig. 2B). This effect was detectable already at 24 h (21% reduction as compared to 29% in the experiment without peptoid) and persisted throughout the experiment (45% reduction as compared to 68% at 96 h after the onset of salt stress). For stringent salt stress (150 mM NaCl), proliferation was almost completely suppressed, leaving only 8.2% (at 96 h) of the activity seen for untreated control (Fig. 2A). Here, the pretreatment with 2 µM of plant PeptoQ restored some of the proliferation with 14.7% (at 96 h) compared to the untreated control (Fig. 2B). From the time courses of proliferation, doubling times were estimated based on an exponential growth model (Fig. 2C). In the absence of salt stress, cell number doubled once a day independently of presence or absence of the peptoid. For moderate salt stress, doubling increased to 39 h in the absence of the peptoid, while in presence of the peptoid, the delay was milder (30 h). For stringent salt stress, doubling became virtually arrested in absence of the peptoid (with a hypothetical time of 330 h required to complete one cycle), while in presence of the peptoid, the doubling was slowed down strongly as well, but significantly less (81 h).

**Figure 3:**
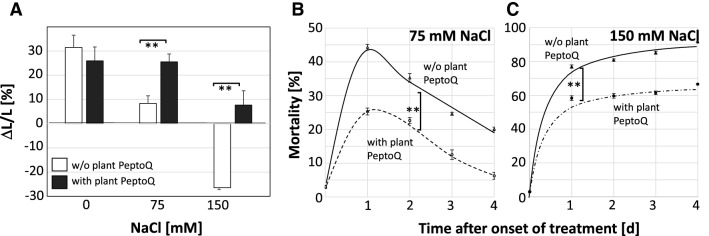
Effect of the plant PeptoQ on salt-induced inhibition of cell expansion and salt induced mortality in tobacco BY-2 cells. (**A**) Relative elongation during the expansion phase (days 3 to 7 after subcultivation) in controls (0 mM NaCl), under moderate (75 mM NaCl), and under severe (150 mM NaCl) salt stress. (**B**,**C**) Time courses of mortality in absence or presence of plant PeptoQ (2 µM) under moderate (**B**), or stringent (**C**) salt stress. Data represent mean values and standard errors of three independent experimental series. **Indicate differences significant at P ≤ 0.01 based on a Student’s *t* test.

We wondered, whether the mitigation of salt stress would also concern the expansion phase between day 3 and day 7 after subcultivation, when the cells enlarge their central vacuole (Fig. 3A). In the absence of the peptoid, relative growth rate decreased drastically already for moderate salt stress (at 75 mM NaCl, less than 30% residual growth as compared to 0 mM NaCl). For stringent salt stress, the values became even negative, meaning nothing else than that the cells shrank, presumably because the osmotic potential in the medium was more negative than that of the protoplast. In presence of the peptoid, the decline of cell expansion was clearly compensated: at 75 mM NaCl, the same growth rate was observed as in the non-stressed controls, meaning that the cells were able to fully compensate the negative osmotic potential of the medium. Even at 150 mM NaCl, a residual expansion of around 30% was maintained (i.e. a level comparable to that seen for 75 mM NaCl in the absence of peptoid). Thus, application of 2 µM of plant PeptoQ fully compensated the impact of moderate salt stress (75 mM NaCl) upon cell expansion, and even for stringent salt stress (150 mM NaCl) allowed for a partial mitigation. As compared to the effects seen on cell proliferation under salt stress, cell expansion seemed to be more responsive to the peptoid treatment.

Arrested proliferation in response to salt stress is usually followed by cell death. Therefore, we followed cell mortality in response to salt stress over 96 h using the Evans Blue Dye Exclusion assay. Under moderate salinity stress (75 mM NaCl, Fig. 3B), in the absence of the peptoid, cell mortality first increased sharply to more than 40% at 24 h, but decreased subsequently over time to 20% (96 h), since the surviving cells continued to proliferate, while the dead cells were not able to do so. While this temporal pattern was also seen in presence of 2 µM of plant PeptoQ, the amplitude of the mortality response was strongly reduced: here, the peak of mortality at 24 h was only 25%, and dropped to 6% at 96 h, which means nothing else than that these cells had fully returned to the viability seen prior to salt stress. For stringent salt stress (150 mM NaCl, Fig. 3C), the cells were not able to recover viability, at least not during the considered time interval of 96 h. In the absence of the peptoid, more than 80% of the cells had died within the first day after addition of salt, and the death toll increased further during the subsequent days reaching to more than 90% (at 96 h). While plant PeptoQ (2 µM) was not able to suppress this salt-induced mortality, it did significantly reduce its amplitude. Even 96 h after the onset of salt stress, still one third of the cells had remained alive (mortality was 67%).

Summarising, application of plant PeptoQ (2 µM) significantly improved the resilience of tobacco BY-2 cells to salinity. Under moderate salt stress (75 mM NaCl), cell expansion and viability were fully preserved whereas cell proliferation was preserved only partially. Even under stringent salt stress (150 mM NaCl), a partial mitigation was observed. Based on these physiological effects, cells of the extreme glycophyte tobacco were shifted, by treatment with plant PeptoQ, to tolerance levels as seen in the marginal halophytes such as barley and date palm^39^.

### Plant PeptoQ improves oxidative homeostasis under salt stress

**Figure 4:**
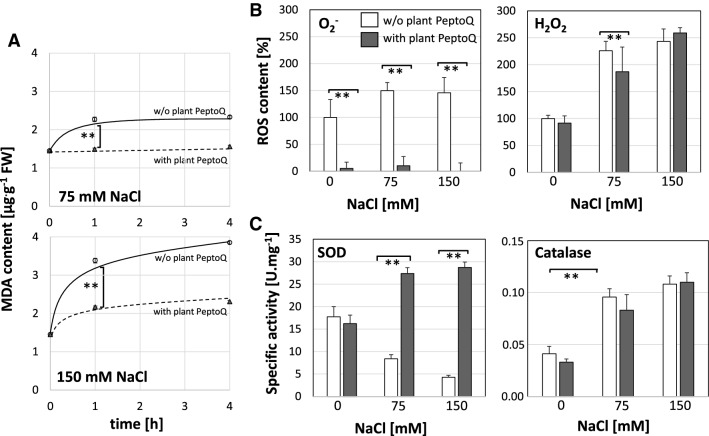
Effect of the plant PeptoQ on oxidative balance. (**A**) Content of malonedialdehyde (MDA) under moderate (75 mM NaCl), and stringent (150 mM NaCl) salt stress in WT BY-2 cells without (black curve) and with (broken curve) plant PeptoQ pretreatment. (**B**) Accumulation of intracellular Reactive Oxygen Species (ROS) in salt stressed non-transformed BY-2 cells without (white square) and with (black square) plant PeptoQ pretreatment. (**C**) Superoxide dismutase (Mn-SOD) and catalase (CAT) activities in absence (white square) and presence (black square) of plant PeptoQ pretreatment. Data represent mean values and standard errors of three independent experimental series. **Indicate differences significant at *P* ≤ 0.01 based on a Student’s *t* test.

The level of lipid peroxidation can be used as the readout for oxidative degradation of membranes^40^ and measured using the product malondialdehyde (MDA) as readout. To investigate, whether plant PeptoQ can compensate the oxidative effects of salt stress on the tobacco BY-2 cells, cells were exposed at the onset of cell expansion (i.e. at day 4 after subcultivation) to salt stress (75 and 150 mM NaCl) either with or without pretreatment with 2 µM of plant PeptoQ, and then sampled for MDA determination at 1 and 4 h after treatment along with a negative control not challenged by salt. MDA content increased in a dose-and time-dependent manner to more than double of the salt-free control, when 150 mM NaCl was applied (Fig. 4A, lower graph). This increase was strongly (by more than 75%) suppressed by plant PeptoQ. For 75 mM NaCl, the peptoid was even able to suppress the salt-induced increase of MDA steady-state levels completely (Fig. 4A, upper graph).

To understand the observed lipid peroxidation monitored by MDA, intracellular ROS level were investigated using the non-fluorescent dihydrorhodamine 123 (DHR 123), which is converted to the green fluorescent rhodamine 123 upon oxidation, mainly by superoxide (O_2_^**∙**−^). The intensity of fluorescence increased progressively, both, in a dose- and a time-dependent manner, in salt stressed BY-2 cells. However, the green fluorescence was quite significantly reduced in salt stressed BY-2 cells pretreated with 2 µM of plant PeptoQ, irrespective of the administered level of salinity, and incubation time (Fig. 4B). Even for stringent salt stress (150 mM NaCl), the suppression of ROS was almost complete. When the cells were observed at high magnification, the fluorescence was not evenly distributed across the cytoplasm, but accumulated in vesicular structures that according to size and subcellular location (e.g. preferentially near the nucleus) resembled mitochondria. The attempt to double-visualise these ROS-generating structures simultaneously with mitochondria using MitoTracker Green was not successful, because the two dyes interfered optically. The patterns seen for MDA are therefore mirrored by the patterns for intracellular ROS formation. This salt induced ROS formation is associated with particular organelles that might be mitochondria (although conclusive evidence for this statement is still lacking).

To get further insight into intracellular levels of specific ROS species, we determined steady-state levels of superoxide (using the Nitroblue Tetrazolium Assay) and hydrogen peroxide (using Ferrous Oxidation with Xylenol Orange Assay) at 4 h after onset of salt stress. In the absence of the peptoid, the steady-state levels of superoxide increased somewhat (by 50%) as compared to the control, albeit not significantly (Fig. 4B, left). However, the peptoid strongly suppressed the accumulation of superoxide (by around 90%) and prevented any salt-induced increase. It should be noted that the superoxide levels were already reduced in the absence of salt. In contrast, at high salt stress (150 mM NaCl), the peptoid did not have any effect on the steady-state levels of hydrogen peroxide (Fig. 4B, right) that increased significantly (to around twice the resting level) under salt stress. Nevertheless, at moderate salt stress (75 mM NaCl), the peptoid caused a slight but significant decrease in hydrogen peroxide level (Fig. 4B, right). We wondered, whether the reduced superoxide levels observed after peptoid pretreatment already prior to the addition of salt would be reflected in changes of mitochondrial morphology, but we could not detect any significant change after visualising mitochondria with MitoTracker Green (Suppl. Fig. S3).

### Plant PeptoQ stimulates SOD activity and expression of the mitochondrial SOD

In order to identify the mechanisms behind the observed effects of peptoid and salt stress on the steady-state levels of intracellular ROS, we measured the specific activities of superoxide dismutase (SOD) and catalase (CAT) as central enzymatic components of intracellular oxidative homeostasis. While in absence of the peptoid, SOD activity decreased to less than a third of the control when salt stress was administered (Fig. 4C, left) but there was a significant increase (by 80%), when salinity was accompanied by treatment with plant PeptoQ. It should be noted that the SOD activity was not increased by the peptoid, when no salt stress was applied, which is in contrast to the superoxide levels that were strongly suppressed by the peptoid even in the absence of salt (compare Fig. 4B,C, left, grey bars for 0 mM salt). In contrast to the effect of plant PeptoQ on SOD activity, no effect of the peptoid was seen, when catalase activity was measured (Fig. 4C, right), although this activity increased significantly under salt stress (around twofold).

**Figure 5:**
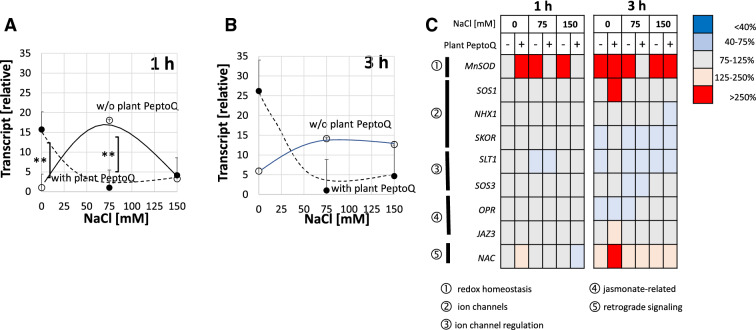
The relative expression of manganese superoxide dismutase (Mn-SOD), mitochondrial gene, at earlier (1 h) (**A**) and later (3 h) (**B**) time points in salt stressed non-transformed BY-2 cells without (black curve) and with (broken curve) plant PeptoQ pretreatment. (**C**) The expression of all the other salt-stress related genes in salt stressed BY-2 cells both with (+) and without (−) plant PeptoQ pretreatment. Data represent mean values and standard errors of three independent experimental series. **Indicate differences significant at *P* ≤ 0.01 based on a Student’s *t* test.

Since the peptoid causes a strong and specific increase of SOD activity (Fig. 4C, left) while at the same time suppressing intracellular steady-state levels of superoxide [the substrate of this enzyme, (Fig. 4B, left)], followed by a reduced lipid peroxidation under salt stress (Fig. 4A), we wondered, whether this activity increase was linked with modulated expression of SOD. We measured steady-state transcript levels after 1 h (Fig. 5A), or after 3 h (Fig. 5B) either in the absence of the peptoid, or after pretreatment with 2 µM of plant PeptoQ for two hours to ensure that the peptoid had fully accumulated in the mitochondria. To ensure comparability, the value at 1 h in absence of the peptoid was used as reference for all conditions. In the absence of plant PeptoQ, moderate salt stress (75 mM NaCl) had induced the transcript level more than 15-fold already 1 h after the addition of NaCl (Fig. 5A), while stringent salt stress (150 mM NaCl) was only inducing by a factor of 3. When measured at 3 h to see a later time point (Fig. 5B), also for 150 mM NaCl, the steady-state transcript level had increased to around 15-fold, and the value for 75 mM NaCl remained at the level seen after 1 h. Interestingly, the peptoid by itself (i.e. in the absence of salt stress) was inducing by around 15 and 25-fold at 1 and 3 h respectively, and this induction was suppressed almost completely under salinity (both 75 and 150 mM NaCl). This suppression was seen for both time points. The comparison with the enzymatic activity of SOD (Fig. 4C) shows that the steady-state transcript levels measured at 3 h were decreased, while the SOD activity measured at 4 h was increased in presence of 2 µM of plant PeptoQ. Thus, accumulation of transcripts and activity of the enzyme encoded by these transcripts, were modulated in the opposite manner.

To judge, whether these strong modulations of steady-state transcript levels were specific for Mn-SOD, or a rather general feature of salinity-related gene expression, we measured the transcripts for relevant ion channels (*SOS1* as channel extruding sodium through the plasma membrane, NHX1 as channel responsible for sequestration of sodium in the vacuole, and *SKOR* as channel involved in sodium–potassium homeostasis), regulators for ion channels (*SOS3* as calcium-dependent regulator of SOS1, and *STL1*, a phosphate regulating sodium–potassium homeostasis), jasmonate-related genes (*OPR* as key enzyme for the peroxisomal synthesis of jasmonic acid, *JAZ3* as salinity-related jasmonate-responsive regulator), and *NAC* as readout for retrograde signalling from mitochondria to the nucleus. Generally, with two exceptions, the vast majority of these transcripts did not show strong modulations (Fig. 5C) neither in response to salinity, nor to the peptoid. Interestingly, the sodium extruder SOS1 showed a threefold increased level in response to the peptoid in the absence of salt stress (Suppl. Fig. S1A). This increase was not seen in presence of salt and it was also not seen for the earlier time point (1 h). This means that SOS1 shows a regulation pattern that was similar to that of Mn-SOD, albeit developing more slowly and also to a much lower amplitude. The other transcript that stuck out, was a tobacco homologue of AtNAC13, which for simplicity is termed *NAC* (Suppl. Fig. S1F): By peptoid treatment, it was first significantly, by 40%, reduced, but later induced, independently of the respective concentration of NaCl. Based on a phylogeny constructed on Arabidopsis and tobacco NAC sequences, this gene seemed to be the closest homologue of ANAC013 (Suppl. Fig. S2), a factor shown to be involved in retrograde signalling of oxidative disbalance^21^.

In addition to the genes mentioned above, additional salt stress-related genes; namely, JAZ1 and JAZ2 (salinity-related jasmonate-responsive regulators), and HKT1 and HAK1 (high-affinity K^+^ transporters) had been investigated in preparatory studies by semiquantitative PCR (SQ-PCR) using the primers mentioned in Suppl. Table S1. However, since no significant responses were detected with respect to the expression of these genes, they were not pursued further.

### Plant PeptoQ does not improve ionic balance under salt stress

**Figure 6:**
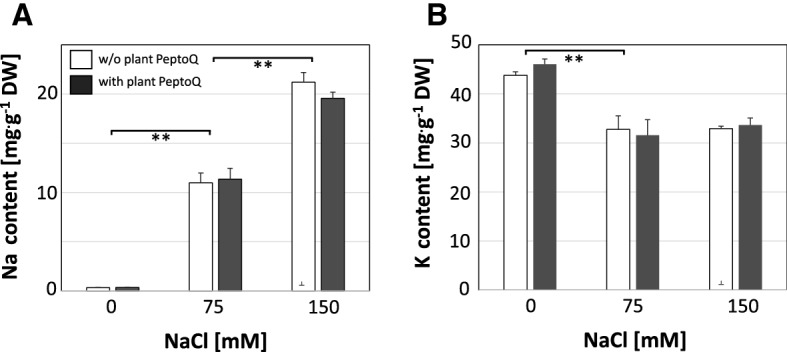
Effect of the plant PeptoQ on salt-induced uptake of sodium (**A**) and potassium (**B**) in non-transformed tobacco BY-2 cells in controls (0 mM NaCl), under moderate (75 mM NaCl), and under stringent (150 mM NaCl) salt stress in absence (white bars) or presence (grey bars) of plant PeptoQ (2 µM). Ion content is given relative to dry weight. Data represent mean values and standard errors of three independent experimental series. **Indicate differences significant at *P* ≤ 0.01 based on a Student’s *t* test.

The damage caused by salt stress is partially due to ionic stress, such as elevated levels of sodium ions, accompanied by depletion of potassium. We determined therefore, the cellular contents for both ions after treatment with 0, 75 and 150 mM NaCl either in absence or presence of 2 µM of plant PeptoQ pretreatment (Fig. 6). As expected, salt stress caused a dose dependent increase in the content of sodium ions (Fig. 6A). This increase was very strong and not mitigated by the peptoid. The uptake of sodium was accompanied by a significant decrease of potassium ions (Fig. 6B). This decrease (around 30% of the initial value) was already saturated for 75 mM NaCl and was not accentuated further for stringent salt stress (150 mM NaCl), although sodium uptake was doubled as compared to moderate salt stress (75 mM NaCl). However, also for potassium, there was no effect of peptoid treatment. Thus, ionic balance is strongly perturbed under salt stress, and this perturbation is not mitigated by plant PeptoQ.

### Plant PeptoQ partitions jasmonate synthesis towards OPDA

**Figure 7:**
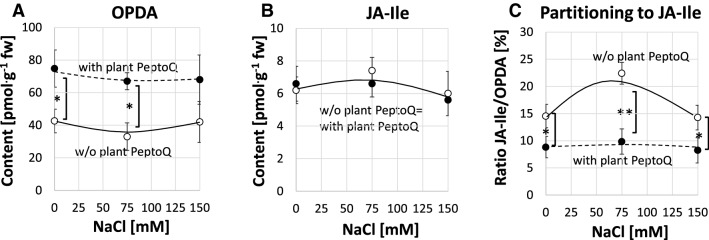
Effect of salt stress on 12-oxo-phytodienoic acid (12-OPDA) and Jasmonic acid-isoleucine (JA-Ile) levels as well as on the ratio between JA-Ile and OPDA (%). (**A**) The level of 12-OPDA in salt stressed non-transformed BY-2 cells with (broken curve) and without (black curve) plant PeptoQ pretreatment (**B**) The level of JA-Ile was almost the same in both plant PeptoQ pretreated and non-pretreated salt stressed BY-2 cells. (**C**) JA-Ile/OPDA (%) in salt stressed BY-2 cells without (black curve) and with (broken curve) plant PeptoQ pretreatment. Data represent mean values and standard errors of three independent experimental series. **Indicate differences significant at *P* ≤ 0.01, *at *P* ≤ 0.05 based on a Student’s *t* test.

Salt stress leads to activation of jasmonate synthesis and signalling, which improves adaptation, if appropriately down-modulated, but can initiate salinity-induced necrosis, if constitutively active^41^. We compared, therefore, the levels of the precursor *cis* (+)12-oxo-phytodienoic acid (OPDA), jasmonic acid (JA), and the final product JA-Ile in salt stressed cells with or without pretreatment with 2 µM of plant PeptoQ after 3 h of salt stress. While JA was not detectable in none of the samples, OPDA and JA-Ile accumulated to measurable amounts (Fig. 7). The steady-state levels of OPDA did not respond to NaCl (Fig. 7A). However, they were significantly increased in presence of the peptoid. This increase was constant (around 35 pmol/g fw), independently of the respective concentration of NaCl. For JA-Ile, the levels were not significantly changed by the peptoid (Fig. 7B). Again, salt stress did not cause any significant change either. However, for moderate salt stress (75 mM NaCl), the content of JA-Ile was slightly (by 15%) increased over the resting level, albeit not significantly. This mild increase was not seen for the higher concentration of salt, nor in presence of the peptoid. Since OPDA is not only a precursor of active jasmonate, but a signal by itself, we plotted the molar ratio between JA-Ile and OPDA, to see, whether salt stress or peptoid treatment repartitioned the balance between these two signalling molecules (Fig. 7C). Here, a significant shift of the biosynthetic pathway from JA-Ile towards OPDA was seen after peptoid treatment, while in the absence of the peptoid, more JA-Ile per OPDA was formed. This channeling of the pathway towards OPDA was significantly more accentuated for 75 mM NaCl, but faded, if the concentration of salt was raised further.

## Discussion

In our previous work^36^, we have developed a construct, plant PeptoQ, composed of a peptoid mediating mitochondrial import, and a functional cargo that mimics coenzyme Q10. We were able to show that this construct was efficiently and specifically accumulating in mitochondria of walled tobacco BY-2 cells and improved their resilience against salinity stress. For cell expansion, this recovery was more pronounced, and, for moderate salt stress, even complete (Fig. 3), while cell proliferation was more sensitive and rescued only partially (Fig. 2). In the current work, we addressed the mechanism, by which plant PeptoQ mitigates salt stress. We found that plant PeptoQ improved oxidative balance under salt stress, evident from reduced steady-state levels of malonedialdehyde and superoxide, which were correlated with increased activity of superoxide dismutase (Fig. 4). The improved resilience was not caused by a better ionic homeostasis, because intracellular sodium increased, and intracellular potassium decreased to the same extent as in the absence of the peptoid in response to salt stress (Fig. 6). However, the steady-state levels of Mn-SOD transcripts were rapidly and strongly upregulated in response to plant PeptoQ in the absence of salt stress (Fig. 5A). This pattern is considered as specific, because the majority of salt-stress related genes (with exception of mild effects seen for the sodium extruder *SOS1*, the retrograde-signalling factor *NAC*, and the jasmonate signalling factor *JAZ3*) did not show significant responses to salt stress. These surplus Mn-SOD transcripts were then consumed under salt stress, which, together with the high enzymatic activity of SOD indicate that the peptoid induces a higher pool of transcripts that are then efficiently converted to protein under challenge. In the absence of the peptoid, Mn-SOD transcript levels were low, but increased in response to salt stress. This increase was more efficient for moderate salt stress (75 mM NaCl) but developed more sluggishly under stringent salt stress (150 mM NaCl). In other words, the pretreatment with 2 µM of plant PeptoQ activated adaptive gene regulation in an anticipative manner. We pursue the working hypothesis that this “pre-adaptation” provides a time gain which is decisive for survival under salt stress. In the following, we will explore several implications of this working hypothesis.

### Is plant PeptoQ modulating retrograde signalling?

Chloroplasts and mitochondria originated from independent prokaryotic organisms, but most of their genes have been either lost or transferred to the nucleus of their eukaryotic host. The proteins encoded by these transferred genes have to be imported by virtue of specific signal peptides. However, both organelles have still retained some genes, for instance, for components involved in electron transport across the inner membranes, or components involved in organelle gene expression^42,43^. The retention of a residual gene set in the organellar genome has attracted considerable attention. One of the most plausible explanations for this phenomenon has been given by the colocation for redox regulation of gene expression (CoRR) hypothesis (reviewed in^44^). Because, in both organelles, free electrons are transported across a membrane in the presence of oxygen, or partially reduced derivatives of oxygen, any disbalance would lead to fatal consequences, which means that there has been a strong selective pressure towards rapid and efficient redox homeostasis, which is not compatible with a regulatory system, where the organelle has first to launch retrograde signaling to the nucleus, transcriptional activation, and then posttranslational import of the respective gene product. Nevertheless, redox homeostasis in plant mitochondria is secured beyond the CoRR mechanism and involves retrograde signalling as well, conveyed by redox-active components, such as ATP, acetyl-CoA, NAD^+^, and glutathione (reviewed in^45^), which shows, how vital it is for a plant cell to safeguard redox balance against perturbations by biotic or abiotic stress. While retrograde signalling from the plastids has been intensively studied from the 1970s (a historical review is given in^46^; for a recent update on the molecular components see the comprehensive review by^47^), plant mitochondrial retrograde signaling is far less understood. However, disruption of mitochondrial electron transport and subsequent accumulation of ROS has been shown as triggers for retrograde signalling^43,48^. Still, the pathway that conveys the information about the perturbed functional status of mitochondria to the nucleus leading to a specific transcriptional response had remained enigmatic over years (reviewed in^49^). Based on mutant approaches, several members of the NAC family of transcription factors, such as NAC13^21^, for a recent review (see^50^), or NAC17^22^ had been identified as crucial component for mitochondrial retrograde signalling. These proteins are integrated in the ER, but can be cleaved by a Rhomboid type protease, such that the N-terminal domain can be imported into the nucleus. Since mitochondria are transported along actin and since actin^51^ also is important for ER^52^ mitochondrial contact, leakage of ROS through the mitochondrial permeability pore is expected to initiate this signal.

At the moment, the molecular nature of the signal that transfers information from the disturbed mitochondria to the transcription factors through the ER remains unknown. However, several studies indicate that mitochondrial ROS (especially superoxide ions) are responsible in relaying such information from the dysfunctional mitochondria to the downstream transcription factors^53,54^. Interestingly, retrograde signalling is not only deployed by excess of mitochondrial superoxide, but as well, when steady-state levels of superoxide drop below normal (for a recent review see^55^). In our study, salt stress caused increased ROS generation (Fig. 4B), impaired SOD activity (Fig. 4C), but increased Mn-SOD transcripts (Fig. 5). This correlation would be consistent with a model, where Mn-SOD upregulation is the consequence of disturbed redox balance in the mitochondria. In response to pretreatment with plant PeptoQ, steady-state levels of Mn-SOD transcripts were strongly upregulated (Fig. 5), while resting levels of superoxide were extremely low (Fig. 4B), as it would be expected, if the elevated transcripts result in enhanced accumulation of protein. This effect is not seen for hydrogen peroxide (Fig. 4B) supporting Mn-SOD as specific target for the peptoid effect. Interestingly, the specific activity of SOD is not altered by the peptoid pretreatment (Fig. 4C), but increases during salt stress, while the steady-state transcript levels for the Mn-SOD transcript decrease strongly (Fig. 5). These data suggest that the peptoid pretreatment stimulates transcription of Mn-SOD, and that this elevated pool of transcripts is then mobilised, once the mitochondria are challenged by salt stress. This scenario would indicate that posttranscriptional regulation becomes active during salt stress, but also that transcriptional activation of Mn-SOD prior to salt stress contributes to the mitigating effect of plant PeptoQ. To achieve this transcriptional activation, a signal has to be conveyed from the mitochondria, where plant PeptoQ accumulates, towards the nucleus, where the Mn-SOD gene is encoded. When this retrograde signaling is mediated by specific NAC proteins that are proteolytically cleaved during the process^21,22^, meaning that the signalling molecule is consumed during signalling, one would expect that the corresponding genes would be upregulated in the long term to replenish the pool of the consumed NAC protein. The finding that the putative tobacco homologue of AtNAC13 is upregulated after prolonged incubation with plant PeptoQ (Suppl. Fig. S1F) is consistent with this hypothesis. Thus, our data support a scenario, where this peptoid activates retrograde signalling from mitochondria to the nucleus and, thus, induces the accumulation of Mn-SOD transcripts. Upon salt stress, this elevated pool is recruited to fuel elevated SOD activity, mitigating the oxidative disbalance caused by the excess of sodium ions.

### Beyond retrograde signalling—a role for post-transcriptional regulation of mitochondrial SOD?

The strong upregulation of steady-state transcript levels seen for Mn-SOD after treatment with 2 µM of plant PeptoQ prior to the onset of salt stress (Fig. 5A) would be expected to lead to elevated SOD activity resulting in reduced superoxide levels and concomitantly increased hydrogen peroxide levels. These implications are definitely not observed. Neither does the peptoid cause any elevated SOD activity in the absence of salt (Fig. 4C, left-hand graph, 0 mM points), nor are hydrogen peroxide levels increased (Fig. 4B, right-hand graph, 0 mM points). The only implication, which is met by the experimental data, is a drastic (by 90%) reduction of superoxide levels in presence of the peptoid (Fig. 4B, left-hand graph, 0 mM points). If superoxide levels are reduced, while SOD activity is not increased, there must be a non-enzymatic component involved. This non-enzymatic component might be plant PeptoQ itself since its hydroxylated ring confers a very strong superoxide-scavenging property^56^. What remains to be explained is, why the massive (already 15-fold after 1 h) increase of transcripts does not culminate in an increase of SOD activity. This discrepancy calls for a role of post-transcriptional and even post-translational regulation. In fact, several mechanisms such as phosphorylation, nitration, methylation, glutathionylation, acetylation and metal incorporation (reviewed in^57^) are known that control mitochondrial SOD beyond retrograde signaling.

Using the mitochondrial Alternative Oxidase as paradigm, the role of local superoxide in activation of retrograde signalling was tested in rice^58^ by expressing SOD in different subcellular compartments before inducing superoxide by methyl viologen. Only when the SOD was overexpressed in the mitochondria, but not in other compartments, did this overexpression attenuate the effect of oxidative stress on Alternative Oxidase expression. Thus, Mn-SOD activity, mitochondrial homeostasis (functionality) and mitochondrial retrograde signalling seem to be intimately linked. As one mechanism, release of oxidised peptides has been proposed (reviewed in^59^). Alternatively, peroxynitrite, resulting from the reaction of superoxide with mitochondrial nitric oxide, can inactivate SOD by specific nitration^60^.

A third mechanism runs through a miRNA shown to regulate the stability of SOD transcripts in cytoplasmic and plastidic SODs^61^. The abundance of the responsible miR398 is decreased under oxidative stress, such that SOD transcripts can accumulate to higher levels. Whether this mechanism can also act on Mn-SOD, is not known. The strong induction of transcripts for Mn-SOD (Fig. 5) by the peptoid would be expected, when the peptoid can downmodulate the steady-state levels of miR398, a testable implication of this hypothesis. Still, none of the three mechanisms would explain, how plant PeptoQ by itself can induce SOD transcripts, while these transcripts are not translating into elevated activity prior to the onset of salt stress (Figs. 4B,C).

A fourth regulatory mechanism might play a role here: the activity of SOD can unfold only in presence of the metal co-factor, in case of the mitochondrial SOD, a Mn ion. The association of this metal co-factor with the SOD apo-protein has been addressed in yeast mitochondria^62^ (reviewed in^63^) and shown to occur in the mitochondria themselves with the still unfolded imported apo-protein. The mitochondrial import of manganese would therefore turn into a regulator of SOD activity. If the peptoid would trigger SOD transcription, while not promote manganese import, a strong accumulation of transcripts would not translate into increased SOD activity, which is exactly what we observe (Fig. 4B,C). If manganese import is stimulated in response to salt stress, enzyme activity would increase, which is again consistent with our observations (Fig. 4C).

Whether any of the four mechanisms is acting in the context of plant Pepto Q activity remains to be elucidated. As unifying principle, they would relay information on the enzymatic activity of mitochondrial SOD to the nucleus, which means that they employ retrograde signalling, albeit the cellular responses would be different: either activation of SOD transcription, down-regulation of a miRNA gene, or activation of Mn-import. These mechanisms are not mutually exclusive, but are likely acting in concert, and they all lead to implications that are testable experimentally.

### Does plant PeptoQ deploy anticipative signalling by triggering the hypoxia pathway?

**Figure 8:**
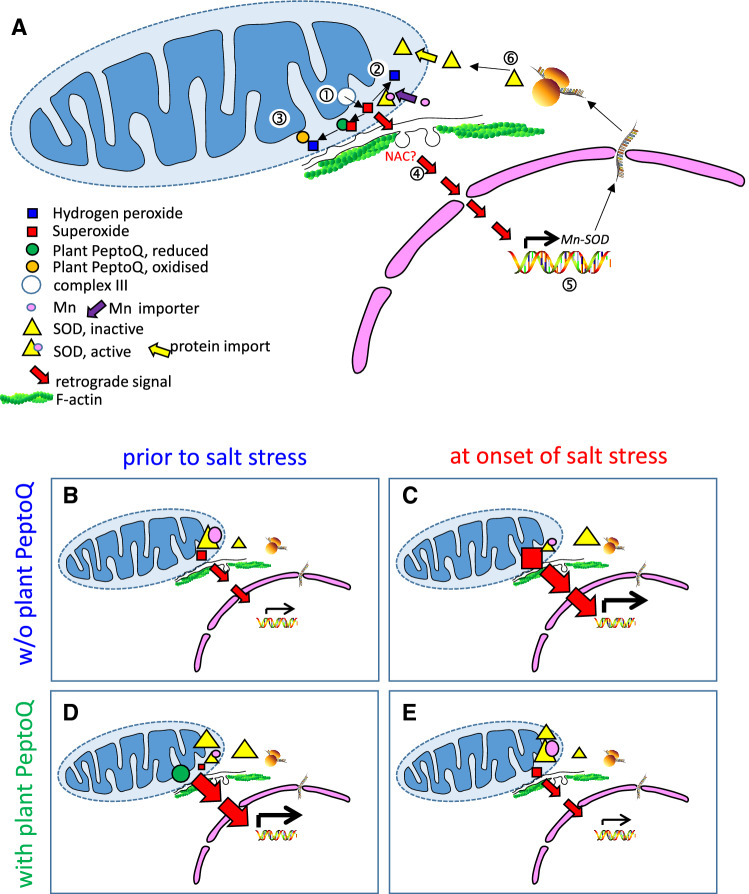
Working model on the mode of action of plant PeptoQ in the context of salt stress. (**A**) Static representation of the model. (**B**–**E**) Implications from the model under different combinations of peptoid pretreatment and salt stress.

The specific and multivariate response patterns seen on the level of transcripts, the level of enzyme activity, and on the level of ROS abundance (Figs. 4, 5) can be explained by a model (Fig. 8A), where plant PeptoQ acts as scavenger of mitochondrial superoxide. Thus, steady-state levels of superoxide (originating from complex III of the mitochondrial electron transport chain, Fig. 8A, ①) dissipate not only by conversion to hydrogen peroxide, catalysed by Mn-SOD (Fig. 8A, ②), but also by direct reduction through plant PeptoQ (Fig. 8A, ③). Thus, under physiological conditions, this additional mechanism of elimination, will reduce superoxide to a level, as it would occur during hypoxia. This deploys hypoxia-related retrograde signalling^55^, possibly involving specific NAC transcription factors (Fig. 8A, ④). Since these NAC proteins would be consumed by proteolytic cleavage, they are expected to be replenished by induction of NAC transcripts, as we actually have observed in our study (Fig. 5C, Suppl. Fig. S1F). This retrograde signalling would culminate in the induction of Mn-SOD (Fig. 8A, ⑤). After translation, the SOD protein is imported into the mitochondria establishing a pool of SOD that would, however, remain inactive, since it is still void of Mn (Fig. 8A, ⑥). This pool of excess SOD is then gradually activated through ongoing Mn import and subsequent complexation into the active holoenzyme. When SOD becomes inactivated under salt stress, for instance by nitration, the additional pool of SOD can be recruited to compensate for this inactivation. Thus, excessive formation of superoxide as it would normally occur under salinity, is prevented, which allows the cells to buffer, at least partially, the negative impact of salt stress.

The implications from this working model under the four combinations derived from two factors (plant PeptoQ, salt stress) and two cardinal states (absence, presence) are met in detail by our observations (Fig. 8B–E). For instance, this model can explain, why pretreatment with 2 µM of plant PeptoQ in the absence of salt stress (compare Fig. 8B,D) leads to the observed (Fig. 4B) very low steady-state levels of superoxide, while, simultaneously, steady-state levels of Mn-SOD transcripts increase more than 20-fold (Fig. 5) without a concomitant increase of SOD activity (Fig. 4C). Likewise, the model can explain, why salt stress, in the absence of the peptoid (Fig. 8C) can induce SOD transcripts (Fig. 5), while SOD activity decreases (Fig. 4C), while, simultaneously, superoxide levels increase (Fig. 4B). Moreover, it can explain, why under salt stress in presence of plant PeptoQ (Fig. 8E) SOD transcript levels drop back to the levels found in cells that neither are exposed to salt stress, nor to peptoid treatment (Fig. 5), while SOD activities increase (Fig. 4C). As a result, the oxidative damage resulting from salinity stress, is efficiently quelled, as evident from the observed suppression of MDA-levels in presence of plant PeptoQ (Fig. 4A).

In summary, the mitigation of salt-induced stress by pretreatment with 2 µM of plant PeptoQ can be explained by triggering the hypoxia pathway leading to anticipative stress signalling.

Although the current work has focused on Mn-SOD as central enzymatic antioxidant, it should not be forgotten that also non-enzymatic antioxidants can help to re-install oxidative balance. However, these compounds accumulate at a slower pace and, thus, act to stabilise the oxidative balance, once it has been re-installed. For instance, a study on salt stress in grapevine cells^64^, showed that the stilbene resveratrol, the major non-enzymatic antioxidant in grapevine, needs around 10 h to accumulate, which is much later than the response of SOD activity found in the current study.

## Outlook: what is the role of plastids?

Salt stress not only represents a challenge for mitochondrial redox homeostasis, but also impairs the performance of plastids. Even in non-photosynthetic cells as tobacco BY-2, plastids are present. Although their inner membranes are reduced, the plastids of suspension cells are metabolically active, and trigger, in response to salt stress, the synthesis of the plant stress hormone jasmonic acid^,^^65,66^_._ Jasmonate signalling clearly responds to peptoid treatment. On the one hand, the transcripts of the jasmonate response factor JAZ3 are upregulated (by 50%) in response to plant PeptoQ in the absence of salt stress (Suppl. Fig. S1H). On the other hand, the jasmonate biosynthesis pathway is partitioned towards the precursor OPDA, on cost of the final signal jasmonate-isoleucine (Fig. 7C). This is interesting, because this precursor is normally exported from the plastids into peroxisomes, where it is converted to jasmonic acid. In the meantime, OPDA has been recognised as signal of its own virtue, triggering a separate signalling pathway^67^. One relevant target of OPDA is the induction of 3′-phosphoadenosine 5′-phosphate (PAP), which is one of the secondary metabolites involved in plastid retrograde signaling^68^. Thus, modulation of redox homeostasis in the mitochondria through plant PeptoQ bears also on retrograde signalling of plastids, leading to the question, how the two organelles can communicate. An exciting possibility are the recently discovered peroxules, filamentous outgrowths of peroxisomes that are formed under stress and get into touch with mitochondria^69^. The peroxisomes as organelles that can interact with both, mitochondria and plastids, seem to be important players in this inter-organelle crosstalk. The partitioning between OPDA and jasmonic acid signalling depends on the enzyme OPDA-reductase. This peroxisomal protein converts OPDA imported from the plastid and represents the first committed step of the jasmonate branch of the pathway. We have, therefore, overexpressed the salinity-related rice OPR7 (OsOPR7) gene in wild type BY-2 cells and have already observed that these cells are significantly more tolerant to salt stress as compared to the non-transformed wild type. Analysis of this cell line with respect to its response to plant PeptoQ should provide new insights into mitochondria-plastid signalling. A possible link with plastids can also be inferred from the fact that salt stress also activates accumulation of abscisic acid, whose synthesis (as that of jasmonates) initiates in the plastid. In fact, a comparative study of two grapevine lines contrasting in salinity tolerance^65^, showed that abscisic acid accumulated to higher levels in the tolerant line, however, downstream of OPDA and JA-Ile accumulation. Additional questions to be addressed in future studies is the anticipative accumulation of SOD protein in the organelles. This can be addressed by specific antibodies raised against recombinantly expressed Mn-SOD, which would allow for Western blotting and in-situ labelling by immunofluorescence. Published records on antibodies that are specific for specific subtypes of SOD^70–72^ indicate that this strategy should be feasible. Moreover, our model implies that, in addition to Mn-SOD, other elements of redox balance might be regulated by the peptoid. In this context, Alternative Oxidase (AOX) is of interest, since its overexpression has been found to improve salt tolerance in Arabidopsis^73^. A further specific marker gene would be succinate dehydrogenase^74^, proposed as a major source for ROS-production in plant mitochondria. Similar to Mn-SOD, this gene is located on the nuclear genome and, thus, regulation, will depend on retrograde signalling.

It seems that the communication between the oxidative organelles and the nucleus plays a crucial role for stress tolerance of plant cells. Plant PeptoQ provides a promising tool to dissect this communication in a dynamic manner.

## Materials and methods

### Cell lines and cell cultivation

Suspension cell lines of tobacco (*Nicotiana tabacum* L. cv Bright Yellow-2^75^ were grown in liquid medium containing 4.3 g/L Murashige and Skoog (MS) salts (Duchefa Biochemie, The Netherlands), 30 g L^−1^ sucrose, 200 mg L^−1^ KH_2_PO_4_, 100 mg L^−1^ (myo)-inositol, 1 mg L^−1^ thiamine, and 0.2 mg L^−1^ 2,4-D, pH 5.8. At weekly intervals, 1.5 mL of stationary cells were inoculated into a 30 mL Erlenmeyer flask with fresh medium and shaken in the dark at 26 °C on a KS260 basic orbital shaker (IKA Labortechnik, Germany) at 150 rpm. Stock BY-2 calli were maintained on media solidified with agar [0.8% (w/v)] and subcultured monthly.

### Cell density determination

Non-transformed BY-2 cells were treated with 0, 75 and 150 mM NaCl after pretreatment with or without 2 µM of plant PeptoQ for 2 h. Cells were counted daily (from day 0 till day 4), in triplicate, using Fuchs-Rosenthal hematocytometer (0.2 mm depth) to determine the maximum cell density and doubling time (Td). Cell number was calculated using the formula: Number of Cells/mL = $\frac{Total \;Number \;of \;Cells}{{Number \;of \;squares}} \times DF \times 5,000$(^76^with minor modification) where DF represents dilution factor. On the other hand, based on the time courses for cell density and the assumption of first order kinetics:

$$\frac{dn}{{dt}} = k \cdot n$$

where n and k represent number of cells and the time constant of exponential growth respectively, we have the natural logarithm ln(n(t)) = ln(n(t = 0)) + kt where the slope K could be approximated by linear regression and the natural logarithm should follow a straight line. Then, doubling time τ (= duration of the cell cycle) could be estimated from the estimated k value based on the following equation: ln (2·n(t = 0)) = ln(n(t = 0)) + kτ as τ = ln (2)/k.

### Measurement of cell length and width

Non-transformed BY-2 cells were treated with 0, 75 and 150 mM NaCl after pretreatment with or without 2 µM of plant PeptoQ for 2 h to measure cell length and cell width. Then, cells at day 3 (exponential phase) and day 7 (stationary phase) after subcultivation/treatment were imaged using an AxioImager.Z1 Apotome microscope (Zeiss, Jena, Germany) with a 20 × objective by a digital image acquisition system with a cooled digital CCD camera (AxioCamMRm; Zeiss) controlled by AxioVision Software 4.8 (Zeiss). Cell width and length of the long cell axis were measured using the AxioVision software Rel. 4.8 (Zeiss) from MosaiX images taken. Data represent standard errors and mean values from more than 500 individual cells collected from 3 independent experiments.

### Determination of viability

To determine cell viability, 0.5 µl aliquots of 0, 75 and 150 mM NaCl treated non-transformed suspension cultured BY-2 cells after pretreatment with or without 2 µM of plant PeptoQ for 2 h were collected at 24, 48, 72, and 96 h after subcultivation/treatment. To remove the medium, each sample was transferred into custom-made staining chambers^77^, and then cells were stained with the membrane-impermeable dye Evans Blue (2.5% w/v, dissolved in distilled water) and incubated cells and dye together for 3–5 min^78^. The Evans Blue was eliminated by washing three times with double distilled water. In dead cells, owing to the breakdown of the plasma membrane, Evans Blue can enter, stain the interior of the cell blue. Thus, those cells that accumulated Evans Blue were considered dead. Cells were mounted on a slide and viewed under Axioskop microscope (Zeiss, Jena, Germany), equipped with a 32 × long distance objective (Zeiss Neofluar, Jena, Germany), and a digital CCD camera (AxioCam MRm). The number of dead and living cells was counted, and percent cell death was calculated as the ratio of the number of dead cells over the total number of scored cells. For each independent treatment, at least 3,000 cells were counted in three independent experiments.

### Visualisation of intracellular ROS

To examine the salt induced ROS production, non-transformed BY-2 cells were treated with 0, 75 and 150 mM NaCl after pretreatment with or without 2 µM of plant PeptoQ for 2 h (at day 3–4 after subcultivation) and placed under continuous shaking. Sampling was done at 10 min, 2 and 4 h after treatment by taking 200 µl of BY-2 cells, suspending them in 800 µl phosphate-buffered saline (PBS) and followed by treatment with a cell-permeable fluorogenic probe reporting oxidative burst^79^ dihydrorhodamine 123 (DHR 123, final concentration 10 µM) at each sampling time. After 30 min incubation, cells were washed 3 times using pre-warmed PBS at 37 °C and resuspended in 1 ml PBS. Changes of the fluorescent signal were followed over time under an AxioImager Z.1 microscope (Zeiss, Jena, Germany) using the filter set 38 HE (excitation at 470 nm, beamsplitter at 495 nm, and emission at 525 nm), a 20 × objective and a constant exposure time of 300 ms.

### Determination of lipid peroxidation

The product of lipid peroxidation, malondialdehyde (MDA), indicator of oxidative burst was measured using a reaction between MDA and 2‐thiobarbituric acid (TBA) according to^80^ with minor modification as follows: 4 days old non-transformed BY-2 cells were treated with 0, 75 and 150 mM NaCl after pretreatment with or without 2 µM of plant PeptoQ for 2 h and samples were collected at 1 and 4 h by removing the medium using vacuum pump and about 0.1 g cells were put in 2 ml Eppendorf tube and frozen in liquid nitrogen. Then the combined cell fresh weight and steel bead was recorded, and cells were shock frozen again in liquid nitrogen, and then homogenized with steel beads (Tissue Lyser, Qiagen/ Retsch, Germany) in standardized manner (twice 30 s at 25 Hz), and frozen in liquid nitrogen. Then 1 ml of sodium phosphate buffer 10 mM (pH 7.4) was added to each sample, homogenized and centrifuged for 4 min at 8000*g*. Then 200 µl of the supernatant from each treatment was added to the respective already prepared reaction mixture containing 100 µl of 8.1% (w/v) Sodium Dodecyl Sulfate (SDS), 750 µl of 20% (w/v) acetic acid (pH 3.5), 750 µl of 0.8%(w/v) aqueous 2‐thiobarbituric acid (TBA) and 200 μl of Milli‐Q water. For the blank, identical reaction mixture was used where the 200 µl supernatant was replaced with the sodium phosphate buffer. Both reaction mixtures were mixed very well and incubated at 98 °C for 1 h. Then the mixtures were cooled at room temperature and centrifuged at 8000*g* for 5 min and absorbance was measured at 535 nm (specific signal) and 600 nm (background). The MDA concentration in the supernatant was determined from the difference in absorption (A535–A600 nm) using a molar extinction coefficient of 155 mM^−1^ cm^−1^ and fresh weight^81^.

### Measurement of superoxide, hydrogen peroxide, and activities of antioxidant enzymes

In order to determine the intracellular levels of reactive oxygen species and the activities of antioxidant enzymes, 0.1 g of both 2 µM of plant PeptoQ pretreated and non-pretreated salt stressed non-transformed suspension cultured BY-2 cells were shock frozen in liquid nitrogen and then lyophilised. Then, the lyophilisate was homogenised in 50 mM phosphate buffer (pH 7.0) supplemented with 1 mM EDTA, 1 mM polyvinylpyrolidone (PVP), and 0.05% v/v Triton X, followed by centrifugation of the homogenate at 10,000*g* for 20 min at 4 °C. Finally, the resulting supernatant was used for determination of ROS-levels and antioxidant enzymes activities. Superoxide ions were measured by using the nitroblue tetrazolium method^82^, hydrogen peroxide was determined by the Xylenol Orange assay^83^. The activity of superoxide dismutase (SOD, EC 1.15.1.1) was determined by the nitroblue tetrazolium scavenging assay^84^, recording superoxide scavenging through the absorbance at 560 nm and calculating enzyme activities based on an extinction coefficient of 12.8 mM^−1^ cm^−1^. Catalase (CAT, EC 1.11.1.6) activity was determined based on hydrogen peroxide scavenging^85^ making use of absorbance at 240 nm by hydrogen peroxide estimating the concentration based on an extinction coefficient of 40 mM^−1^ cm^−1^ in a mixture of 0.5 ml extract and 2.5 ml of buffered substrate (6 mM H_2_O_2_ in 100 mM sodium phosphate buffer, pH 6.8)^86^_._

### Measurement of cellular Na^+^ and K^+^ content

Non-transformed BY-2 Cells were treated with 0, 75 and 150 mM NaCl after pretreatment with or without 2 µM of plant PeptoQ for 2 h at day 4 after subcultivation and incubated on a shaker at 150 rpm. Samples were collected at 1 and 3 h by removing the medium using Büchner funnel fitted vacuum pump, and then dried in an oven at 80 °C for 3 days. Cells were digested according to^87^ with slight modification after determining the dry weight of the samples: Digestion tubes (Gerhardt, UK) were used to mix the dry cells of each technical and biological replicates with 5 ml of concentrated nitric acid (HNO_3_) and then followed by 24 h room temperature incubation along with 6 and 24 h interval vertexing. Samples were boiled in a water bath at 105 °C for 2 h, and after cooling, distilled water was added into each sample in order to obtain a final and adjusted volume of 10 ml which is vortexed accordingly. Eventually, flame atomic absorption spectrometry (AAnalyst200, Perkin Elmer) in an air-acetylene flame (Institute of Applied Geosciences, Karlsruhe Institute of Technology) was used to measure the Na^+^ and K^+^ contents. To prepare blank samples, 5 ml concentrated nitric acid was added into an empty digestion vessel and processed in the same way as above. Three independent technical and biological replicates were taken into consideration in calculating cations concentration with reference to the dry weights.

### OPDA, JA, and JA-Ile endogenous level

In the quantification of endogenous OPDA, JA and JA-Ile levels, standardized ultraperformance liquid chromatography–tandem mass spectrometry (UPLC–MS/MS)-based method was used using [^2^H_5_] OPDA, [^2^H_6_] JA and [^2^H_2_] JA-Ile according to^88^.

### RNA extraction and quantitative real-time PCR

Total RNA was isolated from 0, 75 and 150 mM NaCl treated non-transformed BY-2 cells after pretreatment with or without 2 µM of plant PeptoQ for 2 h (samples were collected at 1 and 3 h from three independent biological repeats) using the InnuPREP plant RNA kit (Analytik Jena AG, Jena, Germany) according to the manufacturer’s instructions. The cDNA synthesis was performed from 1 µg of total RNA extracts using the M-MuLV cDNA Synthesis Kit (NEB), with Oligo(dTs) (Thermo Fisher, Germany) according to the manufacturer’s instructions. Quantitative real-time PCR (qRT-PCR) was carried out on a CFX Touch real-time PCR system (Bio-Rad, Munich, Germany) detection system according to the manufacturer’s instructions with the following cycler conditions: 3 min, 95 °C, 39 × (95 °C for 15 s, annealing at 60 °C for 40 s). Details on the primer sequences and accession numbers for genes of interest and reference genes are listed in Suppl. Table S1. To compare the transcript levels between different samples, the 2^−∆∆Ct^ method was used, the difference in the cycle threshold (Ct) values between the endogenous control genes (elongation factor 1 α (GenBank D63396) and ribosomal protein L25 (GenBank L18908) and target gene was calculated^89^. Data are given relative to the values obtained in control cells that had neither been treated with the peptoid, nor with salt.

## Supplementary information

Supplementary information

## Data Availability

All data generated and analysed in the current study are available from the corresponding author on reasonable request.
