# Supplementary material for: A mitochondria-targeted coenzyme Q peptoid induces superoxide dismutase and alleviates salinity stress in plant cells
Source: Sci Rep. 2020 Jul 14;10:11563. doi: 10.1038/s41598-020-68491-4 (PMC7360622; doi:10.1038/s41598-020-68491-4)
Supplement: Supplementary file 1 — Supplementary information [file 41598_2020_68491_MOESM1_ESM.pdf]

A Mitochondria-Targeted Coenzyme Q Peptoid Induces Superoxide Dismutase and Alleviates Salinity Stress in Plant Cells

Kinfemichael Geressu Asfaw[1], Qiong Liu[1], Xiaolu Xu[1], Christina Manz[1], Sabine Purper[1], Rose Eghbalian[1], Stephan W. Münch[2,4], Ilona Wehl[2,4], Stefan Bräse[2,4], Elisabeth Eiche[6], Bettina Hause[7], Ivan Bogeski[3], Ute Schepers[2,5], Michael Riemann[1], Peter Nick[1]

[1]Molecular Cell Biology, Botanical Institute, Karlsruhe Institute of Technology (KIT), Fritz-Haber-Weg 4, D-76131 Karlsruhe, Germany

[2]Institute of Organic Chemistry, Organic Chemistry I, Karlsruhe Institute of Technology (KIT), Fritz-Haber-Weg 6, D-76131 Karlsruhe, Germany

[3]Molecular Physiology, Institute of Cardiovascular Physiology, University Medical Center, Georg-August-University, 37073 Göttingen, Germany

[4]Institute of Biological and Chemical Systems-Functional Molecular Systems (IBCS-FMS), Karlsruhe Institute of Technology (KIT), Hermann-von-Helmholtz-Platz 1, D-76344 Eggenstein-Leopoldshafen, Germany

[5]Institute of Functional Interfaces (IFG), Karlsruhe Institute of Technology (KIT), Hermann-von-Helmholtz-Platz 1 D-76344 Eggenstein-Leopoldshafen, Germany

[6]Institute of Applied Geochemistry (AGW), Geochemistry and Economic Geology Group, Karlsruhe Institute of Technology (KIT), Adenauerring 20b, D-76131 Karlsruhe, Germany

[7]Department of Cell and Metabolic Biology, Leibniz Institute of Plant Biochemistry, Weinberg 3, D-06120 Halle (Saale), Germany

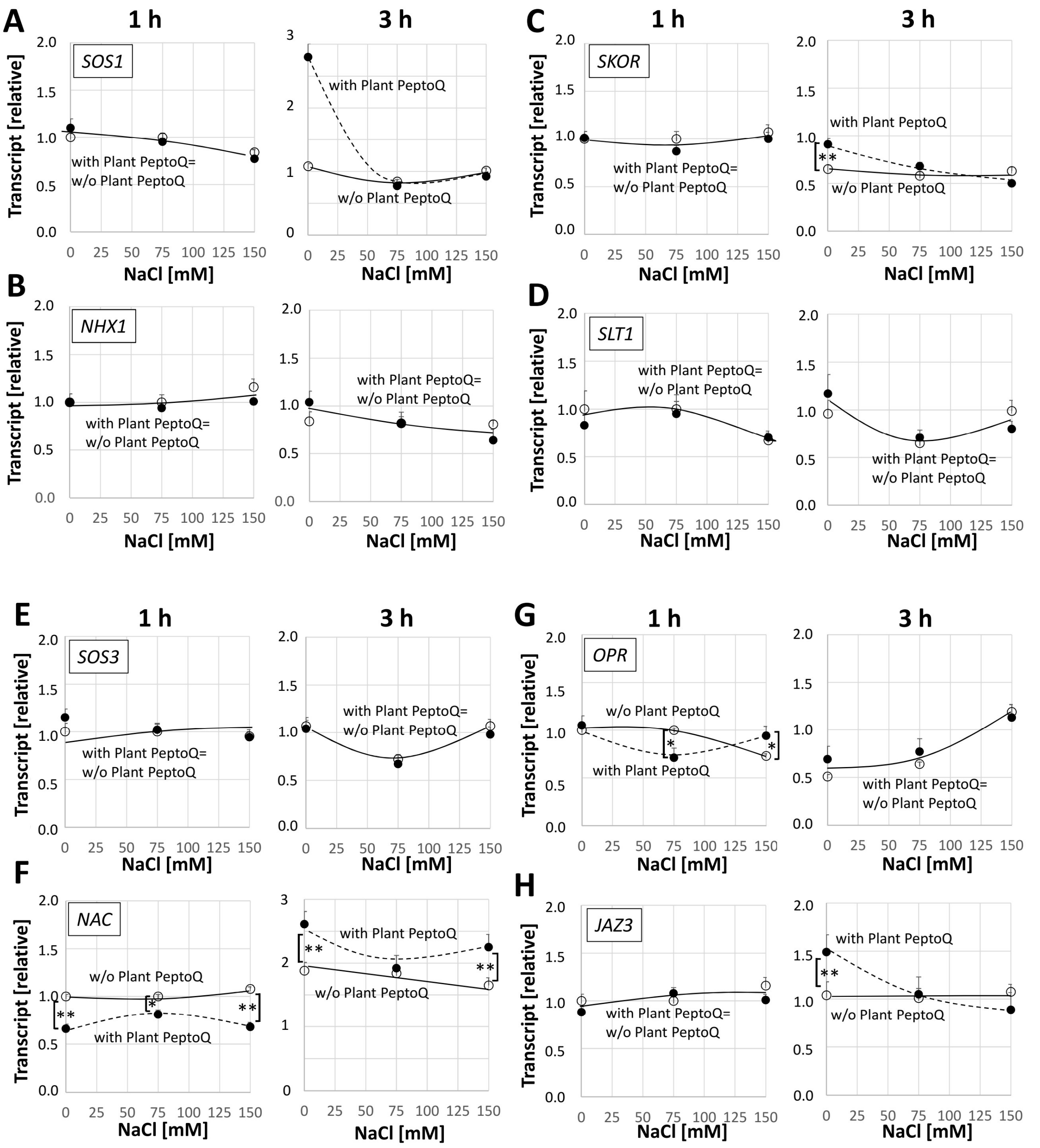

Supplementary Figure S1. Expression of different salt-stress related genes. **A-H** Effect of salt stress on the expression of eight genes in non-transformed WT BY-2 cells without (black curve) and with (broken curve) plant PeptoQ pretreatment. Data represent mean values and standard errors of three independent experimental series. ** indicate differences significant at $P \leq 0.01$, *$P \leq 0.05$ based on a student's t-test.

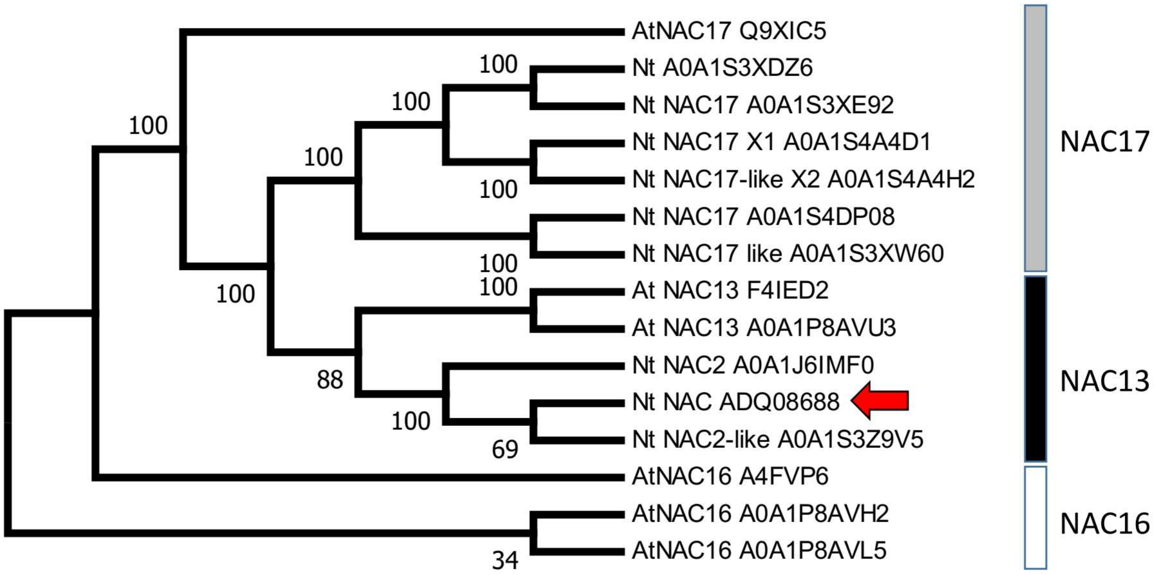

Supplementary Figure S2. The tobacco (*Nicotiana tabacum*), NtNAC gene considered in this study is the homologue of the Arabidopsis (*Arabidopsis thaliana*) NAC13 (ANAC013). The NtNAC (red arrow) is much closely related to Arabidopsis NAC13 unlike its relationship with NAC16 and NAC17.

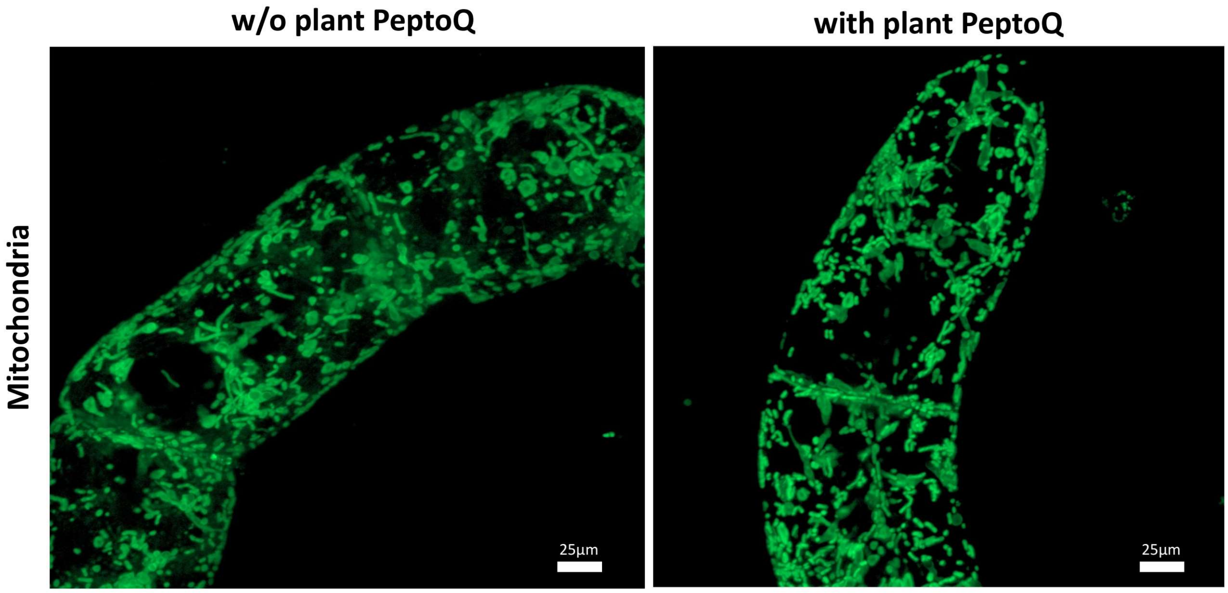

Supplementary Figure S3. Effect of plant PeptoQ on mitochondrial morphology as visualized by staining with MitoTracker Green. Representative confocal images (extended depth of focus projection of z-stacks) show the morphology of mitochondria without plant PeptoQ treatment (left) and with plant peptoQ treatment (right).

Gene name	GenBank accession No.	Forward (5´-3´ prime)	Reverse (5´-3´ prime)
NtEF-1α	D63396	TGAGATGCACCACGAAGCTCTTC	GCTGAAGCACCCATTGCTGGG
NtL25	L18908	GTTGCCAAGGCTGTCAAGTCAGG	GCACTAATACGAGGGTACTTGGGG
NtJAZ1	AB433896	CCAATTGCGAGACGAAATTCACTTAC	CCAAGCCATGCCTTATTTTCCTCATTC
NtJAZ2	AB433897	GCAGCACCTGCTCAACTGACC	GCACCACATTAGGAGGAACGCAACC
NtJAZ3	AB433898	GGATTCCGGTCGATTCGCCG	CCAAGGCTGAGATCTCCAAAGGAAC
NtSOS1	AY383599	GCTCAACGTACACTTCACGG	CCTTGCAACTTCAGCACGAC
NtSOS3	KM658158	CAGAAGAGTGGAAGGAGTTTGC	CTTCAACCTCAGAGCTCATCAC
NtNHX1-Like	XM_016587346	AGGATGCTACTTTCTGCGCC	TGGTTCCTGTTCCGTTGGAG
NtHKT1-Like	XR_001648440	TTCCGATACCCTGAATGGGC	GAGCACTACCAAAACGGCTG
NtSKOR-Like	NM_001326274	TTTATCCCGATGACCGGTGG	AAAGCTTCCTGGGCAATCCC
OsOPR7	XM_015795324	CCAAACGGTGCTGCACCAATATCC	GGTATTTCCGATGCTGCCAGGC
NtSLT1	AF213399	CTTGAAGCGTCGTCCTCAGA	CACCGTTCCTGATCCATCGT
NtHAK1	DQ841950	TTGGACCCAAAGAGTACCGC	GTCCCTCTGAGCGGATGAAT
NtNAC	HQ413134	TTACGCTGGAAAAGCACCCA	ACCCAATCGTCAAGCCTCAA
NtMnSOD	XP_016513256	TCGACACTAACTTTGGCTCCC	GTGGTTTCAATCACCAGGCG

Supplementary Table S1. Overview of primers used in both semiquantitative PCR (SQ-PCR) and quantitative real-time PCR (qRT-PCR).
